# Rethinking bone quality after FDA qualification of total hip BMD as a surrogate endpoint

**DOI:** 10.1016/j.bonr.2026.101968

**Published:** 2026-10-28

**Authors:** Hiroshi Kawaguchi

**Affiliations:** Nadogaya Hospital, Shin-kashiwa, Kashiwa, Chiba, 277-0084, Japan

**Keywords:** Bone quality, Bone mineral density, Surrogate endpoint, Fracture risk, Osteoporosis, Biomarkers, Mechanical competence

## Abstract

• FDA qualified 24-month total hip BMD change as a surrogate endpoint.
• What BMD leaves unexplained should not all be labeled bone quality.
• Bone quality should denote specified, testable skeletal properties.
• Candidate measures require validation matched to their intended use.

## 1 Introduction

For more than three decades, fracture-risk reduction has been the regulatory standard for phase 3 development of osteoporosis therapies. Demonstrating such benefit generally requires several thousand participants followed for several years, making fracture-endpoint trials increasingly costly and difficult (Black et al., 2024). The availability of effective therapies also raises ethical and practical concerns about prolonged assignment of patients at high fracture risk to placebo (Rosen and Khosla, 2010). These barriers may partly explain why no therapeutic class with a distinct mechanism has reached the global market since romosozumab.

On December 19, 2025, the US Food and Drug Administration (FDA) qualified the percentage change from baseline at 24 months in total hip bone mineral density (BMD), measured by dual-energy X-ray absorptiometry, as a surrogate endpoint for phase 3 trials of investigational therapies in postmenopausal women with osteoporosis who are at risk of fracture (US Food and Drug Administration, 2025). Within this defined context, BMD may serve as the efficacy endpoint in place of fracture incidence, potentially enabling smaller and shorter trials.

Regulatory qualification, however, should not be mistaken for biological completeness. BMD does not capture every determinant of skeletal strength or fracture risk; conversely, variation in fracture risk not explained by BMD cannot simply be attributed to “bone quality.” It may reflect skeletal properties not captured by BMD, falls and other loading conditions, discordance between the site of BMD measurement and the fracture site, measurement error, or other unmeasured determinants. In this Perspective, I review what the FDA decision establishes, distinguish bone quality from the broader determinants of fracture, and propose standards for evaluating its candidate components. The aim is neither to reduce skeletal strength to BMD alone nor to dismiss properties beyond BMD, but to clarify what bone quality can and cannot represent across biomechanical research, clinical fracture assessment, and regulatory evaluation.

## 2 What the FDA qualification establishes and what it does not

The scientific foundation for this regulatory transition was provided by the Study to Advance BMD as a Regulatory Endpoint (SABRE), initiated in 2013 as a public-private partnership within the Biomarkers Consortium of the Foundation for the National Institutes of Health. Using individual-participant data from randomized osteoporosis trials, SABRE demonstrated that larger between-group differences in total hip BMD change were associated with greater reductions in vertebral, hip, and nonvertebral fractures (Black et al., 2024, Black et al., 2020). Subsequent analyses established surrogate threshold effects, supported the use of percentage change from baseline at 24 months as the qualified endpoint, and showed that BMD-based conclusions generally converged with fracture outcomes in simulated trials of approximately 500 participants (Eastell et al., 2022; Vilaça et al., 2024; Dufault et al., 2026).

Although SABRE used individual-participant data, surrogacy was evaluated at the trial level: trials with larger active-versus-placebo differences in BMD change tended to show greater fracture reduction (Black et al., 2024; Prentice, 1989). The qualification therefore supports a treatment-group difference in mean BMD change as evidence of likely antifracture efficacy; it does not establish that an individual patient's BMD response predicts whether that patient will fracture or quantify how much of that person's risk is attributable to BMD.

The decision applies within a specified population and regulatory setting (US Food and Drug Administration, 2025). It does not make BMD a comprehensive measure of skeletal strength or a substitute for safety and other clinically relevant outcomes. Moreover, because the evidence derives from mechanisms and trial conditions represented in SABRE, application to a genuinely novel mechanism that reduces fracture risk through effects not reflected by total hip BMD may require additional evidence. These boundaries raise the central question: which determinants not captured by BMD should properly be called bone quality?

## 3 Fracture as an interaction between loading and whole-bone strength

A fracture occurs when the load applied to a bone exceeds its capacity to withstand that load without failure. Applied loading depends on the occurrence and mechanics of falls, impact direction and site, protective responses, muscle forces, and repetitive physiological loading (Komisar and Robinovitch, 2021). The load-bearing capacity of the whole bone is determined by the amount and spatial distribution of bone tissue, external geometry, cortical and trabecular architecture, and tissue-level material properties (Bouxsein, 2005). Falls and applied loading are therefore not components of bone quality. Nor should variation that remains unexplained after accounting for BMD be treated as a biological component of bone, because it may arise from measurement error, differences among study populations, variables omitted from the analysis, or other unmeasured determinants. A low-trauma fracture establishes that the applied load exceeded the capacity of the affected bone, but does not identify whether the principal deficit was low BMD, unfavorable geometry or microarchitecture, reduced tissue-level material properties, or some combination of these factors. Their relative contributions vary with age, skeletal site, and loading condition and cannot be expressed as fixed complementary fractions.

## 4 What should and should not be called bone quality

Bone quality is best retained as an umbrella term for specified structural and material properties that contribute to bone strength but are not directly measured by DXA-derived areal BMD. An influential review proposed four major domains: turnover, collagen-mineral matrix properties, microdamage, and cancellous and cortical architecture or geometry (Burr, 2004). At the whole-bone level, mechanical strength depends on bone size and shape, cortical thickness and porosity, trabecular architecture, and the spatial distribution of bone tissue. Material behavior is influenced by mineral composition, collagen organization and cross-linking, water compartments, microdamage, and noncollagenous proteins (Seeman and Delmas, 2006; Rho et al., 1998; Burr, 2019). Bone remodeling influences both bone structure and tissue material properties. However, circulating bone turnover markers reflect the aggregate activity of bone formation or resorption across the skeleton; they neither localize remodeling to a particular skeletal site nor directly quantify its structural or material consequences or the mechanical strength of an individual bone (Vasikaran et al., 2011).

Aging illustrates why these components must remain distinct. Bone loss, increased cortical porosity, and deterioration of trabecular microarchitecture, including trabecular thinning and loss of connectivity, are accompanied by changes in mineralization, collagen cross-linking, noncollagenous proteins, remodeling rate, water distribution, and microdamage that may reduce ductility and toughness (Burr, 2019; Burr et al., 1997). At the same time, although habitual mechanical loading generally declines with age, loss of muscle strength, balance, and protective responses increases the likelihood of falls and alters their impact biomechanics (Komisar and Robinovitch, 2021). Consistent with this distinction, a prospective study found that both increasing age and decreasing radial bone mass predicted nonspinal fracture, but their relative importance differed by fracture site: age was the stronger predictor of hip fracture, whereas radial bone mass was the stronger predictor of distal forearm fracture (Hui et al., 1988). This site dependence reinforces that age is an integrative risk indicator encompassing both skeletal and extraskeletal changes, rather than a direct measure of bone quality. Thus, the higher fracture probability associated with advancing age at similar BMD values (Kanis et al., 2001) may reflect both age-related deterioration in whole-bone strength—including changes in bone-quality components—and greater exposure or vulnerability to fracture-producing fall loads. The latter represents the loading environment rather than bone quality.

From a biomechanical perspective, a clinically meaningful bone-quality effect should alter a specified mechanical outcome—such as whole-bone stiffness, strength, or energy absorption to failure—relative to bone mass or density (Hernandez and Keaveny, 2006). This shifts attention from treating unexplained variation as a single entity to investigating specific, testable components. Contemporary assessment therefore uses complementary approaches to characterize bone microarchitecture, mechanical behavior, and composition rather than attempting to measure bone quality as a single entity (Wang et al., 2022). Some structural properties can be assessed by quantitative computed tomography, high-resolution imaging, or finite element analysis (Whittier et al., 2020). Matrix composition, microdamage, and tissue toughness remain more challenging to assess in vivo, although emerging methods provide component-specific information. Ultrashort echo-time magnetic resonance imaging can quantify cortical bone water in vivo (Techawiboonwong et al., 2008), and MRI-derived measures of bound and pore water have been associated with mechanical properties relevant to fracture resistance, including toughness (Manhard et al., 2016). More recent human studies suggest that MRI-derived bound and pore water may also provide information relevant to fracture risk beyond areal BMD (Nyman et al., 2023). Spatially offset Raman spectroscopy can also retrieve transcutaneous information on the mineral and organic phases of bone in vivo (Buckley et al., 2015). Transcutaneous measurements in human hands have confirmed that bone spectral information can be detected, although contributions from overlying soft tissue are not completely eliminated (Chen et al., 2021), and the clinical discriminatory value of this approach remains to be established. These approaches assess particular structural or material components rather than integrated bone quality or whole-bone mechanical competence.

The 2001 NIH Consensus Statement remains historically important because it described bone strength as integrating bone density and bone quality and stated that BMD accounts for approximately 70% of bone strength (NIH Consensus Development Panel on Osteoporosis Prevention, Diagnosis, and Therapy, 2001). The statement did not cite a specific dataset or analysis from which this percentage was derived. A subsequent review similarly summarized biomechanical observations indicating that DXA-measured BMD explains approximately 60–70% of the variation in experimentally measured bone strength (Ammann and Rizzoli, 2003). The 70% figure should therefore be understood as an approximate, context-dependent measure of explanatory performance rather than a universal biological partition. The NIH statement did not state that the remaining 30% was bone quality. In mechanical studies, variation not explained by DXA-derived BMD may reflect structural and material properties not captured by DXA, measurement error, differences among skeletal sites and specimens, and differences in which variables were measured and included in the analysis. When fracture risk rather than mechanical strength is the outcome, falls and loading introduce additional determinants. The explicit 70:30 formulation in the 2025 Japanese guideline (Committee for the Development of the Guidelines for Prevention and Treatment of Osteoporosis, 2025) provides a contemporary example of how a context-dependent statistical association may, through repeated citation and simplification, be interpreted as a fixed biological partition. The broader lesson extends beyond any single guideline and concerns the communication and clinical translation of statistical findings. Bone quality should not be presented as a single biomarker or outcome. Claims that a disease impairs or a treatment improves bone quality should specify the property measured and its demonstrated relationship to mechanical competence or fracture.

## 5 From biological component to clinically useful biomarker

Pentosidine illustrates the gap between biological plausibility and clinical measurement. Advanced glycation end-products in bone collagen may impair material properties, making pentosidine a plausible candidate marker (Saito and Marumo, 2010). However, a concentration in blood or urine cannot be assumed to reflect pentosidine accumulation or mechanical behavior specifically in bone.

A meta-analysis reported higher urinary pentosidine in individuals with fractures, but six of the seven studies in its principal analysis were from Japan and much of the evidence was cross-sectional (Shirinezhad et al., 2025). Higher urinary pentosidine was associated with incident clinical fracture in older adults with type 2 diabetes (Schwartz et al., 2009), whereas pentosidine did not independently predict fracture after adjustment for age, prevalent fracture, and total hip BMD in the OFELY cohort (Gineyts et al., 2010). Renal clearance influences circulating and urinary pentosidine concentrations (Miyata et al., 1998), while diabetes, aging, and oxidative stress affect pentosidine formation and accumulation (Saito and Marumo, 2010). Although the Japanese guideline describes pentosidine as a marker of bone-quality deterioration and an independent fracture risk factor (Committee for the Development of the Guidelines for Prevention and Treatment of Osteoporosis, 2025), it is more appropriately regarded as a research biomarker whose bone specificity and incremental clinical value remain unestablished.

These distinctions should remain explicit. Biological plausibility alone does not establish that a marker is specific to bone. An association with fracture does not show that it improves prediction beyond BMD and established risk factors, and improved prediction does not by itself establish clinical utility. Similarly, a treatment-related change in a marker does not demonstrate that the change mediates fracture reduction. Qualification as a surrogate endpoint requires a further level of evidence: for total hip BMD, the FDA relied on trial-level analyses linking between-treatment differences in BMD change to differences in fracture reduction within a defined context of use (Black et al., 2024, Black et al., 2020; US Food and Drug Administration, 2025; Eastell et al., 2022; Vilaça et al., 2024; Dufault et al., 2026). Candidate bone-quality measures should therefore be evaluated and interpreted according to their intended use rather than assigned equivalent status on the basis of mechanistic or observational evidence alone.

## 6 A research agenda for bone quality in the BMD-surrogate era

Translation requires three linked levels of validation. First, the construct and measurement must be explicit: the intended structural or material property, its hierarchical level and relationship to mechanical competence, analytical precision, reproducibility, and anatomical-site relevance should be defined. Measurements from peripheral bone, iliac crest, blood, or urine should not automatically be assumed to represent the hip or vertebral body.

Second, clinical validity must extend beyond association. A candidate should predict incident fracture independently of BMD and established risk factors and provide meaningful incremental information. Relevant heterogeneity by fracture site, age, sex, diabetes, renal function, and treatment should be examined rather than absorbed into a general label of impaired bone quality.

Third, responsiveness, causal relevance, and utility must be distinguished. A marker may change during therapy without mediating fracture reduction. Claims of improvement require reproducible change in the specified property; claims that the change contributes to fracture prevention require mediation or trial-level surrogate evidence. Ultimately, measurement should improve treatment selection, monitoring, or outcomes beyond current assessment (Califf, 2018). Bone quality remains an important research domain, but progress depends on component-specific, falsifiable claims and validation proportionate to their intended use.

## 7 Conclusion

FDA qualification of total hip BMD as a surrogate endpoint is a major regulatory advance, but not evidence that BMD fully captures skeletal strength or fracture risk. Bone quality remains a useful concept when it refers to specified structural or material properties that can be measured and tested; it becomes misleading when treated as a single entity comprising everything left unexplained by BMD. The next step is therefore not to abandon bone quality, but to replace fixed partitions and the assumption that all variation unexplained by BMD constitutes bone quality with component-specific measures validated for mechanical relevance, incremental fracture prediction, treatment responsiveness, and, where claimed, mediation of antifracture efficacy.

## CRediT authorship contribution statement

**Hiroshi Kawaguchi:** Writing – review & editing, Writing – original draft, Conceptualization.

## Declaration of Generative AI and AI-assisted technologies in the writing process

Generative AI (ChatGPT, OpenAI GPT-5.6) was used only to assist with language editing. All writing, scientific content, interpretation, and final approval of the manuscript were performed solely by the author.

## Funding

The author received no financial support for the research, authorship, or publication of this article.

## Declaration of competing interest

The author declares no known competing financial interests or personal relationships that could have appeared to influence the work reported in this paper.

## Data Availability

No data was used for the research described in the article.
